# Effects of Different Levels of Garlic Straw Powder on Growth Performance, Meat Quality, Antioxidant and Intestinal Mucosal Morphology of Yellow-Feathered Broilers

**DOI:** 10.3389/fphys.2022.902995

**Published:** 2022-05-26

**Authors:** Shuang Liao, Liping Liao, Peng Huang, Yanzhou Wang, Siyuan Zhu, Xin Wang, Tuo Lv, Yinghui Li, Zhiyong Fan, Touming Liu, Qian Lin

**Affiliations:** ^1^ Institute of Bast Fiber Crops, Chinese Academy of Agricultural Sciences, Changsha, China; ^2^ College of Animal Science and Technology, Hunan Agriculture University, Changsha, China

**Keywords:** garlic straw powder, yellow-feathered broilers, growth performance, meat quality, antioxidant, intestinal mucosa morphology

## Abstract

The full utilization of garlic straw can partially alleviate shortage of feedstuff and waste of resources. The purpose of this study was to investigate the effects of garlic straw as an unconventional feed on yellow-feathered broilers. 360 28-day-old yellow-feathered broilers were randomly divided into 4 groups with 6 replicates (cage) per group, 15 per cage. The 4 groups were as follows: control group (basal diet) and experimental group I (basal diet supplemented with 3% garlic straw powder), II (basal diet supplemented with 6% garlic straw powder) and III (basal diet supplemented with 9% garlic straw powder). There was no significant difference in the initial body weight of the broilers among groups (*p* > 0.05). The test period was 28 days in total. The experiment results showed that there were no significant difference in the average final weight, ADG, ADFI and F/G among groups (*p* > 0.05). On the one hand, for the breast muscle, the drip loss of experimental group I, II and III were reduced by 17.24% (*p* <0.05), 20.11% (*p* <0.05) and 20.50% (*p* <0.05), respectively, compared with the control group; the redness a* of the experimental groups had a trend of improvement (0.05 <*p* < 0.1). On the other hand, compared with the control group, the redness a* of the experimental group II increased significantly by 23.18% for the leg muscles (*p* < 0.05). Furthermore, compared with the control group, GSH-Px of the experimental group III significantly increased by 21.38% (*p* < 0.05), and SOD of the experimental group I significantly increased by 21.85% (*p* < 0.05). Finally, there were no significant differences in the intestinal villus height, crypt depth, V/C and intestinal wall thickness among four groups (*p* >0.05). In conclusion, dietary supplementation of different levels of garlic straw powder can improve meat quality and antioxidant capacity of yellow-feathered broilers without affecting growth performance and intestinal mucosal morphology.

## Introduction

The sustainability of livestock production systems ensures global food security (Davis and White., 2020). However, as the number of humans and livestock soared, there was inevitably a situation of people and livestock competing for food. Non-conventional feed raw material resources are “a large number of scraps” or “garbage” neglected by the material production sector, including stems of agricultural crops, residues from slaughterhouses, excreta of livestock and poultry, and plant meal (Yin et al., 2019). These resources are widely available, low cost and high feeding value. In fact, it has become a reality to use this “garbage” to solve some problems in agriculture. A prime example is Singapore’s use of technology, such as recycling nutrients from food waste, to address food shortages (Mok et al., 2020). This makes us think, can we also use non-conventional feed raw material resources to solve the issue of insufficient feed resources ?

Garlic straw is the main by-product of garlic. Studies showed that the crude protein content of garlic straw is similar to that of alfalfa hay and the crude fiber content is lower (Lee et al., 2017). Garlic straw is also rich in active functional components, including more than 30 kinds of allicin, organic selenium, superoxide dismutase, enzymes and glycosides (Karangiya et al., 2016; Zhang et al., 2019). Nicastro et al. (2015) highlight potential mechanisms of metabolites of allicin, including decreased bioactivation of carcinogens, antimicrobial activities, and redox modification. To be specific, allicin and other sulfide substances contained in garlic straw can inhibit the proliferation of both bacteria and fungi, including antibiotic-resistant strains like methicillin-resistant *Staphylococcus aureus* (Borlinghaus et al., 2014; Nakamoto et al., 2020). Long-term human intake of animal-derived antibiotics may cause pathogenic bacteria to develop drug resistance, thereby breaking the ecological balance of bacteria in the body (Chen et al., 2019). Feeding garlic by-product to livestock and poultry may maintain the balance of flora to a certain extent, and ultimately benefit humans (Wunderlich et al., 2014; Zhu et al., 2021).

Yellow-feather broiler accounts for more than half of Chinese broiler market, which satisfy consumer preferences regarding flavor in China and some other countries in South-East Asia (Gou et al., 2016). At present, garlic straw has achieved good feeding effect in rabbits (Liu L. et al., 2019), buffalo (Attri et al., 2020), sheep (Lee et al., 2017) and other animals. There is no relevant research on the feeding of garlic straw to yellow feather broilers. Therefore, this study took the high-quality broiler breed yellow-feather broiler as the research object, and explored the application effect of garlic straw as an unconventional feed resource in yellow-feather broiler.

## Materials and Methods

### Garlic Straw Powder Preparation

The garlic straws used in this study were collected from Jinxiang County, Shandong Province, China. Garlic straw powder is made by cutting, drying and pulverizing, and stored in a closed dark room for preparation. After analysis and determination, the conventional nutrient content and apparent metabolizable energy of the garlic straw powder samples used in this study are as follows: dry matter content 88.83%, crude protein 10.81%, crude fiber 25.97%, crude fat 1.40%, crude ash 12.87%, calcium 3.02%, total phosphorus 0.24%, apparent metabolizable energy 1.65Mcal/kg.

### Animals and Experimental Details

All the experiment procedures were conducted in accordance with the Chinese guidelines for animal welfare and approved by the Animal Care and Use Committee, Institute of Bast Fiber Crops, Chinese Academy of Agricultural Sciences. In this study, 360 28-day-old yellow-feathered broilers were randomly divided into 4 groups with 6 replicates (cage) per group, 15 per cage. The 4 groups were as follows: control group (basal diet) and experimental group I (basal diet supplemented with 3% garlic straw powder), II (basal diet supplemented with 6% garlic straw powder) and III (basal diet supplemented with 9% garlic straw powder). The initial weight of chicks was approximately similar for all groups. The whole experiment period was 28 days.

The basal diet was formulated with reference to the nutritional requirements of broilers in NRC (1994) and NY/T33-2004 Chicken Feeding Standard. The raw material dosage and source of the whole feed were kept the same, and the specific composition and nutritional level were shown in Table 1.

**TABLE 1:** Diet formulation and calculated nutrients (as fed basis).

Items	Control	Garlic straw powder supplementation concentration in diets		
		3%	6%	9%
Ingredients, %				
Corn	50.00	50.26	50.96	51.30
Soybean meal	30.52	30.00	29.57	29.04
Rice husk	4.60	3.03	1.56	0.00
garlic straw powder	0.00	3.00	6.00	9.00
Oil	3.43	3.13	2.69	2.35
Wheat bran	7.55	6.90	5.77	5.07
Limestone	1.71	1.47	1.22	0.97
CaHPO4·2H2O	0.74	0.74	0.74	0.76
L- Lys	0.00	0.02	0.03	0.05
98.5% DL- Met	0.15	0.15	0.16	0.16
NaCl	0.30	0.30	0.30	0.30
3% Premix1)	1.00	1.00	1.00	1.00
Total	100	100	100	100
Nutrient levels (%)				
ME (Mcal/kg)	2.85	2.85	2.85	2.85
Crude protein (%)	18.00	18.00	18.00	18.00
Crude fiber	5.71	5.71	5.71	5.71
Calcium (%)	1.00	1.00	1.00	1.00
Total phosphorus (%)	0.61	0.61	0.60	0.60
Available P	0.35	0.35	0.35	0.35
Lysine (%)	0.99	0.99	0.98	0.99
Methionine (%)	0.46	0.46	0.46	0.46
Methionine + cystine (%)	0.76	0.76	0.76	0.75

^a^The premix provided the following (per kilogram of complete diet): vitamin A 12000 IU; vitamin D3 2500 IU; vitamin E 20 mg; vitamin K3 3 mg; vitamin B1 3 mg; vitamin B2 8 mg; vitamin B6 7 mg; vitamin B12 0.03 mg; Pantothenic acid 20 mg; Nicotinic acid 50 mg; Biotin 0.1 mg; Folic acid 1.5 mg; Cu 9 mg; Zn 110 mg; Fe 100 mg; Mn100 mg; Se 0.16 mg; I 0.6 mg. 2) Nutrient levels were calculated values.

This experiment was carried out in Yuanjiang Experimental Base of Hemp Research Institute, Chinese Academy of Agricultural Sciences. First, thoroughly clean and disinfect the coop 1 week before the experiment. Second, a 24-h intelligent light source and ventilator are installed in the broilers house to provide appropriate temperature and ventilation according to the temperature needs of broilers. Third, the broilers are fed different diets according to the experimental design. Broilers have free access to food and water. In addition, daily management and immunizations are carried out in strict accordance with the standard procedures of the broilers farm. Record the actual daily feed intake and observe the behavior and health of the chickens from time to time.

### Sample Collection

On day 28 of the experiment, 6 broilers were randomly selected from each treatment for blood sampling. Blood samples were collected from the jugular vein of the broiler into a vacuum blood collection tube. The whole blood was coagulated in a tube at room temperature and centrifugated at 3,500 rpm for 15 min. The serum samples were separated and stored at −20°C until it was used for the determination of serum indicators After blood collection, the broilers were euthanized by carbon absorption. After cervical dislocation, muscle samples (left pectoral and leg muscles) and intestinal samples (duodenum, jejunum and ileum tissues) were quickly separated from the body in a sterile environment. Muscle samples were assayed rapidly. The middle section of each intestine was about 2 cm, cleaned gently with normal saline, and fixed with 4% paraformaldehyde solution.

### Growth Performance

Body weight of yellow feather broilers was individually measured at the beginning (day 28) and the end of the trial (day 56). Feed intake per cage was recorded daily. The average daily gain (ADG), average daily feed intake (ADFI), and food conversion ratio (FCR) were calculated according to the data from each cage.

### Determination of Meat Quality

The meat colour of 45 min postmortem was, respectively, determined as the L*, a* and b*, the indicators of lightness, redness and yellowness, with a colorimeter (CR-400; Konica Minolta Co., Osaka, Japan). The water loss rate (WLR, using a filter paper press method) and pH values (45 min postmortem) were determined as described previously (Choi et al., 2016). The pH value was measured with a pH meter (Model 340, Mettler-Toledo GmbH, Schwerzenbach, Switzerland). The drip loss was scored based on a suspension method with the size-standardised samples (2.0 cm*1.5 cm*1.5 cm) from the leg muscle and breast muscle that were weighed, suspended in a plastic bag, held at 4°C for 24 h, and thereafter reweighed. Drip loss was expressed as a percentage. And, the samples which tested the cooking loss at 45 min postmortem were bagged in tin foil and immersed in a 75°C water bath for 30 min and cooled at room temperature for 30 min. After cooling to room temperature, the tin foil was opened and free juice was drained. The cooked samples were blotted with a paper towel and weighed. The cooking loss was determined by weighing the meat before and after cooking (Honikel., 1998). The shear force was further measured with the digital tenderness meter (C-LM3B, Northeast Agricultural University, Harbin, China) after measuring the cooking loss to evaluate tenderness. Test speeds were set at 2 mm/s. Data were collected and analysed on the basis of the shear force values to obtain the maximum force required to shear through each sample.

### Antioxidant Indices

The serum and hepatic levels of glutathione peroxidase (GSH-Px), the activities of superoxide dismutase (SOD), catalase (CAT), reduced glutathione (GSH), total antioxidant capacity (T-AOC) and malondialdehyde (MDA) were determined by the commercial kits (Nanjing Jiancheng Bioengineering Institute, Nanjing, China) with an automated fluorescence instrument (Thermo Fisher Scientific). The GSH-Px activity was assayed at 412 nm by quantifying the oxidation rate of reduced GSH to oxidised glutathione (Yin et al., 2013). The activity of SOD was determined at 450 nm by the nitrite coloration method (Liu et al., 2014). Briefly, the CAT activity was determined by incubating sample with a known concentration of H_2_O_2_, which was then measured at 405 nm by the ammonium molybdate method (Sun et al., 2015). The GSH concentration was measured according to the methods as described previously with minor modification (Zhan and Meng, 1989). The T-AOC was determined at 520 nm with the method of ferric reducing ability of plasma (Sun et al., 2015). The MDA concentration was determined at 532 nm by thiobarbituric acid reaction method (Yang et al., 2010).

### Determination of Intestinal Mucosa Morphological Structure

The intestinal samples were cleaned and stained with hematoxylin and eosin (HE) and finally made into paraffin sections using microtome (RM-2235, Leica microsystems AG Co., Ltd., Black Forest, Germany). Microscopes were then used to observe tissue paraffin sections, multiple non-contiguous fields were randomly selected, and representative fields were selected to be imaged. Next, the Mingmei Microscope Digital Measurement and Analysis System V1.6.1 was used to observe and measure, to determine the thickness of the intestinal wall thickness, the villus height, and the crypt depth, and to calculate the ratio of villus height to crypt depth (V/C).

### Statistical Analysis

The data were analysed using one way ANOVA model among four groups in Statistical Packages for Social Science 25.0 (IBM). Orthogonal polynomial contrasts were used to determine the Linear and quadratic responses of different levels of garlic straw powder to growth performance, meat quality, antioxidant capacity and intestinal mucosal morphology of broilers. The broilers selected from each group was experimental unit for the analyses of all the data of the present study. The results were expressed as arithmetic means and SEM. When the main test was significant, the differences among means were further analysed using Tukey–Kramer test for multiple comparison. Differences between means of all groups were considered significant at *p* < 0.05. The *p* values between 0.05 and 0.10 were considered as a trend.

## Results

### Growth Performance

The effects of different levels of garlic straw powder on growth performance of yellow-feathered broilers is presented in Table 2. There were no significant differences in average final weight, ADG, ADFI, and FCR among four groups (*p* > 0.05). No mortality was observed throughout the trial period.

**TABLE 2:** Effects of different dietary levels of garlic straw powder on growth performance in yellow feather broiler (g).

Item	Control	Garlic straw powder supplementation concentration in diets			SEM	*p*-value		
		3%	6%	9%		ANOVA	Linear	Quadratic
Average final weight	1447.57	1464.03	1500.87	1481.23	11.706	0.436	0.204	0.451
ADG	34.58	34.95	36.71	35.37	0.501	0.486	0.373	0.407
ADFI	129.54	122.97	126.00	120.82	1.582	0.237	0.107	0.822
FCR	3.77	3.54	3.43	3.42	0.063	0.155	0.038	0.352

^a,b^Means in the same row with different superscript letters indicate differences (*p* < 0.05).

Abbreviations: ADG, average daily gain; ADFI, average daily feed intake; FCR, food conversion ratio; SEM, standard error of the mean.

### Meat Quality

Table 3 shows the effects of different levels of garlic straw powder on breast muscle of yellow-feathered broilers. Compared with the control group, the drip loss of breast muscle was decreased by 17.24% (*p* < 0.05), 20.11% (*p* < 0.05) and 20.50% (*p* < 0.05) at 3, 6 and 9% garlic straw powder supplemental levels, and showed a significant linear change (*p* < 0.05). The experimental groups supplemented with garlic straw powder tended to increase the redness of breast muscle a* (0.05<*p* < 0.1). In addition, there were no significant differences in shear force, L* and b* of meat color, cooking loss and pH value of breast muscle among four groups (*p* > 0.05).

**TABLE 3:** Effects of different dietary levels of garlic straw powder on breast muscle quality in yellow-feathered broilers (g).

Item		Control	Garlic straw powder supplementation concentration in diets			SEM	*p*-value		
			3%	6%	9%		ANOVA	Linear	Quadratic
Shear force		3.23	2.68	3.05	2.72	0.139	0.458	0.364	0.693
Meat colour	L*	50.92	52.87	50.76	53.80	0.647	0.273	0.261	0.667
	a*	8.00	8.26	9.01	8.66	0.150	0.073	0.035	0.274
	b*	7.71	7.44	7.72	8.08	0.189	0.724	0.440	0.430
Cooking loss/(%)		21.73	21.32	22.19	20.29	0.591	0.729	0.537	0.549
Drip loss/(%)		5.22^a^	4.32^b^	4.17^b^	4.15^b^	0.162	0.048	0.016	0.140
PH value		6.05	6.15	6.01	5.85	0.046	0.151	0.076	0.158

^a,b^Means in the same row with different superscript letters indicate differences (*p* < 0.05).

Abbreviations: L*, brightness; B*, redness; A*, yellowness; SEM, standard error of the mean.

Table 4 shows the effects of different levels of garlic straw powder on leg muscle of yellow-feathered broilers. Compared with the control group, the redness a* of drumsticks in the experimental group II significantly increased by 23.18% (*p* < 0.05). In addition, there were no significant differences in shear force, L* and b* of meat color, cooking loss, drip loss and pH ratio of leg muscle among four groups (*p* > 0.05).

**TABLE 4:** Effects of different dietary levels of garlic straw powder on leg muscle quality in yellow-feathered broilers (g).

Item		Control	Garlic straw powder supplementation concentration in diets			SEM	*p*-value		
			3%	6%	9%		ANOVA	Linear	Quadratic
Shear force		1.59	1.60	1.35	1.38	0.080	0.608	0.254	0.959
Meat colour	L*	48.19	50.28	48.40	48.84	0.938	0.878	0.994	0.682
	a*	15.70^b^	16.57^b^	19.34^a^	17.75^ab^	0.476	0.029	0.025	0.150
	b*	9.06	9.78	10.16	10.30	0.468	0.810	0.361	0.769
Cooking loss/(%)		17.49	17.70	16.04	16.23	0.997	0.920	0.572	0.996
Drip loss/(%)		6.70	5.58	7.00	6.90	0.303	0.332	0.460	0.399
pH value		6.28	6.32	6.34	6.26	0.028	0.745	0.856	0.309

^a,b^Means in the same row with different superscript letters indicate differences (*p* < 0.05).

Abbreviations: L*,brightness; B*,redness; A*, yellowness; SEM, standard error of the mean.

### Antioxidant Performance

Table 5 shows the effects of different levels of garlic straw powder on antioxidant performance of yellow-feathered broilers. Compared with the control group, the serum GSH-Px level in experimental group III significantly increased by 21.38% (*p* < 0.05). At the same time, compared with the control group, the serum SOD level in experimental group I significantly increased by 21.85% (*p* < 0.05). In addition, there were no significant differences in CAT, GSH and T-AOC among four groups (*p* > 0.05).

**TABLE 5:** Effects of different dietary levels of garlic straw powder on serum antioxidant performance in yellow-feathered broilers.

Item	Control	Garlic straw powder supplementation concentration in diets			SEM	*p*-value		
		3%	6%	9%		ANOVA	Linear	Quadratic
GSH-Px/(U/mL)	2062.37^b^	2303.23^ab^	2201.08^b^	2503.23^a^	60.810	0.037	0.013	0.731
SOD/(U/mL)	484.50^b^	590.35^a^	473.48^b^	465.30^b^	18.917	0.031	0.175	0.061
CAT/(U/mL)	0.03	0.02	0.02	0.02	0.003	0.209	0.128	0.187
GSH/(umol/L)	5.40	7.56	7.10	6.64	0.436	0.371	0.418	0.161
T-AOC/(mmol/L)	1.00	1.23	0.96	1.31	0.110	0.674	0.543	0.804
MDA/(nmol/mL)	4.75	3.73	4.41	4.51	0.344	0.800	0.989	0.480

^a,b^Means in the same row with different superscript letters indicate differences (*p* < 0.05).

Abbreviations: GSH-PX, glutathione peroxidase; SOD, superoxide dismutase; CAT, catalase; GSH, glutathione; T-AOC, total antioxidant capacity; SEM, standard error of the mean.

### Intestinal Mucosa Morphological Structure

**FIGURE 1:**
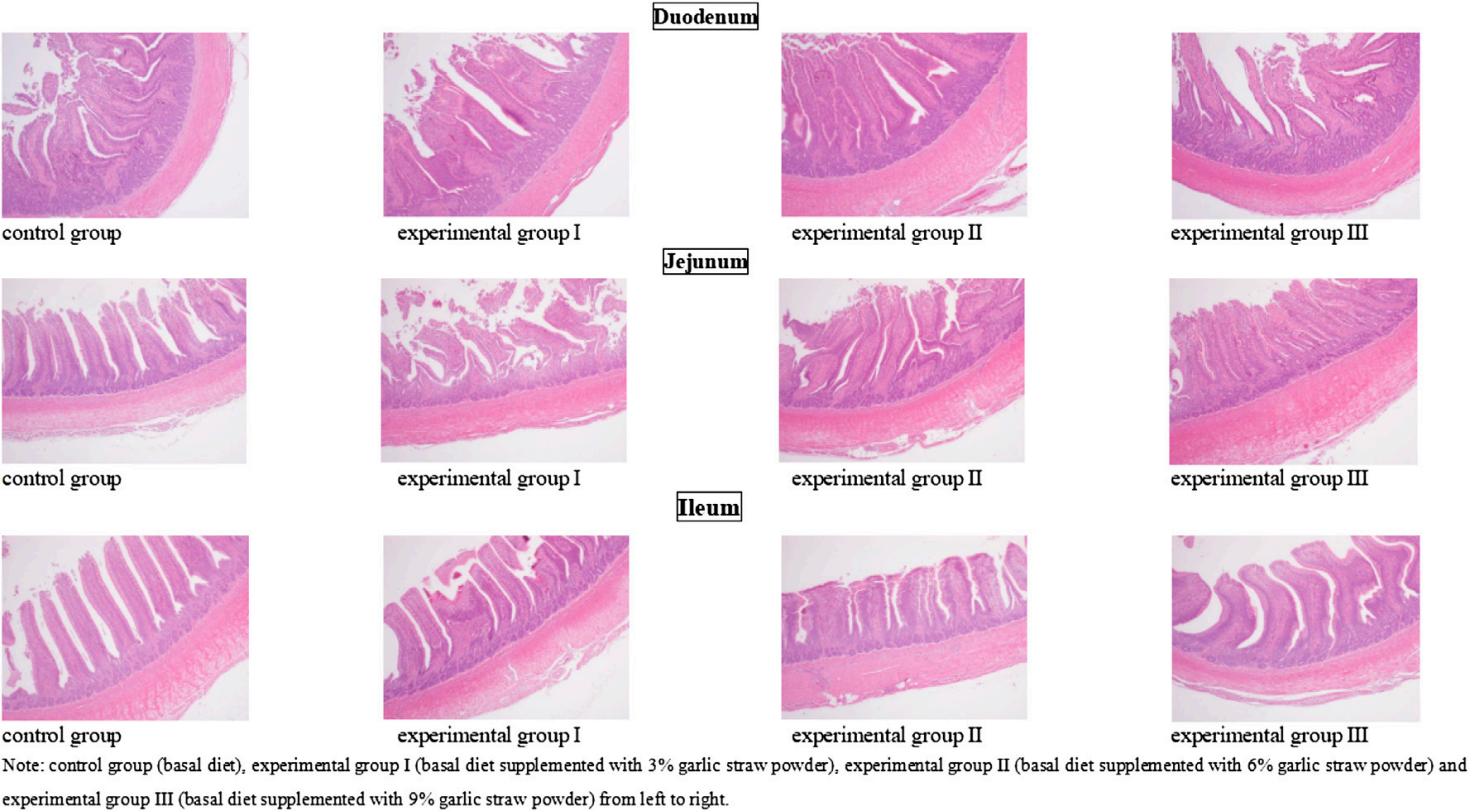
Sections of duodenum jejunum and ileum of yellow-feathered broilers in each group (40×). Note: control group (basal diet), experimental group I (basal diet supplemented with 3% garlic straw powder), experimental group II (basal diet supplemented with 6% garlic straw powder) and experimental group III (basal diet supplemented with 9% garlic straw powder) from left to right.

The effects of different levels of garlic straw powder on intestinal structure of yellow-feathered broilers were shown in Table 6 and Figure 1. There were no significant differences in intestinal wall thickness , villus height, crypt depth and V/C in duodenum, jejunum and ileum of among four groups (*p* > 0.05).

**TABLE 6:** Effects of different dietary levels of garlic straw powder on intestinal morphology in yellow-feathered broilers.

Item		Control	Garlic straw powder supplementation concentration in diets			SEM	*p*-value		
			3%	6%	9%		ANOVA	Linear	Quadratic
Duodenum	Villus height	600.12	605.04	629.53	540.41	13.606	0.130	0.185	0.078
	Crypt depth	115.95	117.87	113.16	103.67	4.426	0.788	0.424	0.603
	V/C	5.25	5.14	5.58	5.24	0.134	0.795	0.778	0.750
	Intestinal wall thickness	229.00	221.95	212.63	189.87	6.702	0.168	0.044	0.505
Jejunum	Villus height	455.08	493.99	460.24	470.86	8.214	0.379	0.981	0.436
	Crypt depth	95.84	102.49	100.56	111.82	2.586	0.178	0.058	0.610
	V/C	4.78	4.83	4.58	4.23	0.103	0.137	0.038	0.304
	Intestinal wall thickness	201.39	223.40	223.89	229.45	5.356	0.352	0.128	0.464
Ileum	Villus height	539.86	521.22	482.89	511.67	14.512	0.635	0.398	0.463
	Crypt depth	99.94	88.92	93.67	94.95	2.976	0.692	0.731	0.365
	V/C	5.42	5.91	5.14	5.40	0.132	0.227	0.461	0.649
	Intestinal wall thickness	195.86	201.06	200.77	187.93	3.646	0.612	0.503	0.274

^a,b^Means in the same row with different superscript letters indicate differences (*p* < 0.05).

Abbreviations: V/C,the villus height/crypt depth; SEM, standard error of the mean.

## Discussion

The addition of herbal extracts and herbs to poultry diets may have some beneficial effects on performance (Abd El-Hack et al., 2018). The multiple effective pharmacological component of garlic straw coincide highly with garlic (Majewski, 2014). It has been reported that the addition of 0.5 and 1.0% garlic in the diet can promote the feeding and improve the growth performance of broilers (Ismail et al., 2021). Studies showed that adding 0.25, 0.50 and 0.75 g/kg garlic powder in the diet promoted the increase of body weight and daily gain of broilers at 21 and 42 days (Ismail et al., 2021). It is speculated that garlic by-products are similar to garlic and can improve the performance of broiler. However, there were no significant differences of average final weight, ADG, ADFI, and FCR among four groups (*p* > 0.05), but no mortality was observed in the whole process in this study. A possible guess is that broilers have a certain tolerance threshold to garlic straw. Garlic and its products have been shown to have a pungent odor and anti-nutrition factors that can reduce the palatability of diets and lead to a decrease in feed intake (Abe et al., 2020; Gu et al., 2021). Some scholars suggest that garlic or garlic powder can be used with other plant to stimulate the digestive enzyme activities of broilers, and then improve the performance and economic benefits of broilers. For example, broilers showed better the weight gain and FCR when both garlic and black pepper were added to the diet (Kirubakaran et al., 2016).

Yellow-feathered broiler is one of the best broiler varieties in China, and its meat quality is valued by consumers (Liu W. et al., 2019). Improving meat quality of yellow-feathered broilers by diet is a common method in breeding industry (Jiang et al., 2018). Previous studies have shown that breast muscle and leg muscle of broilers treated with black garlic extract can significantly improve meat color (Barido et al., 2021). Under the conditions of this study, adding different levels of garlic straw powder tended to increase the a* value of broilers (*p* < 0.05). The color of meat is essentially the form and content of myoglobin (Barido et al., 2021). Choi et al. believed that adding 5% garlic powder can improve a* value of broilers, mainly because its antioxidant components can delay the formation and oxidation of myoglobin (Choi et al., 2010). In addition, this study showed that the drip loss of breast muscle was negatively correlated with the level of garlic straw meal supplementation (*p* < 0.05). Drip loss is closely related to muscle softness, which indirectly reflects human satisfaction with edible quality (Warner et al., 2022). This indicator also indirectly reflects the nutritional value and hygienic quality of broilers. Some studies have shown that increased oxidative damage can impair the tenderness of the meat, thereby reducing the palatability and nutritional value of meat products (Zhang et al., 2013; Hauck et al., 2019). Garlic straw powder may inhibit the oxidation of protein and lipid in broilers by increasing the antioxidant content in broilers, and ultimately improve the tenderness and color of broilers.

Many factors, such as fast growth, high metabolism and intensive production, can produce a large number of free radicals in broilers (Lauridsen , 2019). These free radicals have been proved to induce disease in broilers, resulting in huge economic losses (Clark et al., 2019; Mishra and Jha. 2019). GSH-Px, SOD, and CAT together constitute the main antioxidant enzyme system in broilers, and the level of its content reflects the effect of scavenging free radicals (He et al., 2017). Plant or plant extracts can act on enzymes related to free radicals to indirectly organize the oxidation of free radicals, thereby enhancing the antioxidant capacity of animals (Gladine et al., 2007; Zhang et al., 2020). Previous studies have shown that the addition of garlic powder to the diet has a positive effect on the antioxidant properties of broilers (Ismail et al., 2021). The compound feeding of garlic root powder and Moringa leaf powder also increased the activity of glutathione peroxidase in broilers (Gbore et al., 2020). We speculate that perhaps garlic straw powder also has a similar potential to improve the antioxidant function of yellow feather broilers. In fact, compared with the control group, the serum GSH-Px level of broilers in the experimental group III significantly increased by 21.38% (*p* < 0.05). In addition, the serum SOD level in the experimental group I significantly increased by 21.85% compared with the control group (*p* < 0.05). Similar results were obtained by Locatelli who believed that organosulfur compounds such as allicin had strong anti-radical mechanisms and iron-reducing ability (Locatelli et al., 2017). L-theanine is a water-soluble, non-protein amino acid, mainly found in green tea leaves. L-theanine at 200 mg/kg improved the antioxidant status in broiler blood by increasing SOD, GSH-Px and relative CAT levels (Saeed et al., 2018). Combined with the results of meat quality in this study, it was further verified that adding garlic straw powder can improve the antioxidant capacity of yellow feather broilers.

Intestinal tract is the final site for nutrient digestion and absorption. Intestinal villus height, crypt depth, V/C and intestinal wall thickness indirectly indicate intestinal absorption function and immune function (Mowat and agace, 2014). Most scholars believe that the morphological and structural integrity of small intestine reflects the effective absorption rate of animal nutrients and resistance to related diseases (Solis de los Santos et al., 2005). Studies have shown that the addition of garlic and its by-product derivative (propane thiosulfonate) can improve the growth performance of broilers by protecting the integrity of the intestinal barrier and the composition of related microflora (Ruiz et al., 2015).The possible explanation is that some of the bioactive compounds in garlic can promote the absorption of chyme from the intestine (Hafidh et al., 2011). Previous studies have shown that multi-strain probiotic, citric acid, garlic powder or their combinations can improve the ileal structure of broilers (Elbaz et al., 2021). However, there were no significant differences in villus height, crypt depth, V/C and intestinal wall thickness in duodenum, jejunum and ileum among four groups (*p* > 0.05). We believe that this is related to dosage of garlic straw powder. Previous studies have shown that dietary 1000 mg/kg garlic powder can protect the integrity of intestinal structure of broilers at 21 days of age, while 1000 and 2000 mg/kg garlic powder can significantly protect the integrity of intestinal structure of broilers at 42 days of age (Yang et al., 2021). To sum up. these results indicate that dietary garlic straw powder has no adverse effects on intestinal development.

## Conclusion

In conclusion, garlic straw has no adverse effects on growth performance and intestinal mucosal morphology of yellow-feathered broilers. 3–9% substitution rate of garlic straw powder can improve antioxidant capacity and meat quality.

## Data Availability

The raw data supporting the conclusions of this article will be made available by the authors, without undue reservation.
